# Residents' Dissatisfaction and All-Cause Mortality. Evidence from 74 European Cities

**DOI:** 10.3389/fpsyg.2017.02319

**Published:** 2018-01-09

**Authors:** Ana I. Ribeiro, Sílvia Fraga, Henrique Barros

**Affiliations:** ^1^Epidemiology Research Unit—Instituto de Saúde Pública, Universidade do Porto, Porto, Portugal; ^2^Departamento de Ciências da Saúde Pública e Forenses e Educação Médica, Faculdade de Medicina, Universidade do Porto, Porto, Portugal

**Keywords:** urban health, city planning, European Union, physical environment, health services, socioeconomic environment, community participation

## Abstract

**Background:** About 2/3 of the Europeans reside in cities. Thus, we must expand our knowledge on how city characteristics affect health and well-being. Perceptions about cities' resources and functioning might be related with health, as they capture subjective experiences of the residents. We characterized the health status of 74 European cities, using all-cause mortality as indicator, and investigated the association of mortality with residents' dissatisfaction with key domains of urban living.

**Methods:** We considered 74 European cities from 29 countries. Aggregated data on residents' dissatisfaction was obtained from the Flash Eurobarometer, Quality of life in European cities (2004–2015). For each city a global dissatisfaction score and a dissatisfaction score by domain (environment, social, economic, healthcare, and infrastructures/services) were calculated. Data on mortality and population was obtained from the Eurostat. Standardized Mortality Ratios, SMR, and 95% Confidence Intervals (95% CI) were calculated. The association between dissatisfaction scores and SMR was estimated using Generalized Linear Models.

**Results:** SMR varied markedly (range: 73.2–146.5), being highest in Eastern Europe and lowest in the South and Western European cities. Residents' dissatisfaction levels also varied greatly. We found a significant association between city SMR and residents' dissatisfaction with healthcare (β = 0.334; IC 95% 0.030–0.639) and social environment (β = 0.239; IC 95% 0.015–0.464). No significant association was found with the dissatisfaction scores related with the physical and economic environment and the infrastructures/services.

**Conclusions:** We found a significant association between city levels of mortality and residents' dissatisfaction with certain urban features, suggesting subjective assessments can be also used to comprehend urban health.

## Introduction

Urbanization is probably one of the most important demographic phenomena of our times (Galea and Vlahov, 2005). Currently, about two thirds of the Europeans and more than half of the world population reside in cities (WB, 2014). Thus, more than ever, we must expand our knowledge about how city characteristics affect the health and well-being of the urbanites (Galea et al., 2005). An healthy city is “*one that is continually creating and improving those physical and social environments and expanding those community resources which enable people to mutually support each other in performing all the functions of life and developing to their maximum potential*” (WHO-EUROPE, 1995), which, ultimately, promotes human health and urban sustainability (Portney and Sansom, 2017). This multifaceted definition implies that cities must offer health-supportive physical and socioeconomic environments and adequate access to infrastructures and services, which, according to several conceptual models, represent the main determinants of population health within urban contexts (Galea and Vlahov, 2005). Altering affordances of the physical and social environment can contribute to reduce the stress and unhealthy behaviors associated with urban living, to the restoration of depleted cognitive resources and, by this means, promote a healthy Biosphere (Hartig and Kahn, 2016).

City government plays an enormous role in changing several of these affordances and urban planning can be seen as a form of “preventive medicine” (Corburn, 2015). Indeed, for centuries the most important public health actions started in large urban areas and this trend might accentuate with the increasing decentralization that has brought more power to the local governments (Lawrence, 2013), which deal with a network of city actors/organizations (civil society, corporates, trade unions, informal organizations) and, at same time, cope with the decisions of the central state's agencies (Devas, 2001). To design healthy cities and to motivate planners toward this goal it is crucial to include the civil society, to monitor the current state of population's health, and to take into consideration the residents' opinions about the cities' resources and functioning (Frankish et al., 2002; Hogan et al., 2015). As stated by Burrows and Rhodes in 1998 “we need to move from assuming what is best for people to letting them say what they think would be better” (Burrows and Rhodes, 1998).

Cities, per definition, share some characteristics. Yet, due to distinct geographic situation, size, and cultural and historical background, European cities are very unequal in terms of physical and socioeconomic environments. These specificities might shape the indicators of health and well-being of these cities and, by this means, might create an unequal distribution of health. Health inequalities across European regions have been largely documented (Richardson et al., 2014; Ribeiro et al., 2016), but few studies systematically evaluated the differences in health status across European cities (Gray et al., 2012; Richardson et al., 2017) or explored the determinants of such differences (Richardson et al., 2017). Those determinants are likely to be heterogeneous and difficult to grasp. Despite much attention given to objective measures about the cities environment [pollution (Beelen et al., 2014), greenness (Gascon et al., 2016), socioeconomic deprivation (Marí-Dell'Olmo et al., 2015)], perceptions about the places might also influence and inform on other dimensions of people's health, as they capture subjective experiences of the residents, something that traditional, objectively measured indicators cannot. Subjective assessments capture the “human perception of space” (i.e., place) improving our the understanding of the urban environment and of the population preferences and dislikes (McCrea et al., 2006; Kothencz et al., 2015). Individual perceptions are derived from filtering objective characteristics through standards of evaluation, which depend on past experiences, aspirations, and personal characteristics (John, 1987). By this means, objective attributes become subjective and lead to a certain degree of satisfaction (John, 1987; Amérigo and Aragonés, 1997; McCrea et al., 2006).

A handful of studies, conducted at individual-level, have reported that residents' satisfaction with cities' resources and functioning is related with health outcomes. In 2012, after inquiring over 5550 Taiwanese youth, Shiue found that satisfaction with neighborhood environment was associated with self-rated health (Shiue, 2012). More recently, Hogan and colleagues evaluated the relationship between happiness levels and city environment. After analyzing data from 5,000 adults aged 25–85 years old living in Berlin, Paris, London, New York, and Toronto, they found that younger adult's happiness levels were associated with having easy access to cultural, shopping, transport, parks and sport amenities and the attractiveness of their cities, whereas, among the older participants, it was more strongly associated with the provision of quality governmental services (Hogan et al., 2016). Perceived neighborhood safety and social environment, specifically, have been often associated with self-rated health (Wen et al., 2006; Kim et al., 2013; Assari et al., 2015), health-related quality of life (Parra et al., 2010), mental illness (Polling et al., 2014), and stroke risk (Kim et al., 2013). And, it is important to highlight that some of these studies confirmed that this association remained significant after accounting for objective measures (Wen et al., 2006; Kim et al., 2014) and one have found that perceived measures, rather than objective ones, have a bigger impact over the studied outcomes (Polling et al., 2014).

Residents' ratings about city resources and functioning have been regularly collected at request of European Union through large Europe-wide surveys (EU, 2016) and might constitute a convenient and informative data source to characterize European cities from that point of view. Although some evidence exists that perceived urban characteristics are associated with individuals' health, so far those datasets remain underexplored and underutilized. Thus, the present study aimed (i) to characterize the health status of 74 European cities, using as indicator all-cause mortality, and (ii) to investigate whether mortality levels are associated with the residents' dissatisfaction with five key domains of urban living: physical, social and economic environment, healthcare, and infrastructures/services.

## Materials and methods

### Data

All data was obtained at city-level. A city is a local administrative unit where the majority of the population lives in an urban center of at least 50,000 inhabitants (EUROSTAT, 2016).

Data on residents' dissatisfaction was obtained from the Flash Eurobarometer, “Quality of life in European cities” (EU, 2016). This survey has been conducted since 2004 every 3 years at the request of the Directorate-General for Regional and Urban Policy to get a snapshot of people's opinions on a range of urban issues. Surveys were conducted in 2004, 2006, 2009, 2012, and 2015. The latest survey covered 79 European cities plus four greater cities (greater city is an approximation of the urban centers when this stretches far beyond the administrative city boundaries, EUROSTAT, 2017) and inquired a total of 41,000 citizens.

Although the type and number of items vary by year and country, this survey includes roughly 55 items, covering issues as diverse as employment, environment, housing, transport, culture, city services, and immigration (Table 1).

**Table 1:** Items from the Quality of life in European cities survey included in the creation of the summary scores of resident's dissatisfaction (*n* = 50).

**SERVICES AND INFRASTRUCTURE (*n* = 18)**	
Public transport in the city, for example bus, tram or metro	Outdoor recreation outside/around this city, such as walking, cycling or picnicking
Schools in the city	Minutes per day spent traveling to work/training place
Sports facilities such as sport fields and indoor sport halls in the city	Why don't you use public transport?
Cinemas in the city	Most important in my city: public transport
Cultural facilities such as concert halls, theaters, museums, and libraries in the city	Most important in my city: education and training
Public Internet access such as internet cafes or libraries in the city	Most important in my city: road infrastructure
Internet access at home in the city	State of streets and buildings in my neighborhood
When you contact administrative services of this city, they help you efficiently	Availability of retail shops
This city spends its resources in a responsible way	Public spaces in this city such as markets, squares, pedestrian areas
**SOCIAL ENVIRONMENT (*n* = 9)**	
Foreigners who live in this city are well integrated	Most important in my city: social services
The presence of foreigners is good for this city	You feel safe in this city
Generally speaking, most people in this city can be trusted	You feel safe in the neighborhood you live in
Most important in my city: Urban safety	The public administration of the city can be trusted
Most people in my neighborhood can be trusted	
**ECONOMIC ENVIRONMENT (*n* = 9)**	
In this city it is easy to find a good job	The financial situation of your household
In this city, it is easy to find good housing at a reasonable price	Most important in my city: jobs creation / reduce unemployment
You have difficulty paying your bills at the end of the month	Most important in my city: housing conditions
In this city, poverty is a problem	Your personal job situation
Most important in my city: Unemployment	
**PHYSICAL ENVIRONMENT (*n* = 10)**	
Green spaces such as public parks or gardens	The beauty of streets and buildings in your neighborhood
In this city, air pollution is a big problem	Most important in my city: air pollution
In this city, noise is a big problem	Most important in my city: noise
This city is a clean city	The quality of the air in the city
The cleanliness in the city	The noise level in the city
**HEALTHCARE (*n* = 4)**	
Health care services offered by hospitals in the city	Most important in my city: health services
Health care services offered by doctors in the city	Health care services offered by doctors and hospitals in this city

To obtain an overall picture of the residents' dissatisfaction in each city, a global dissatisfaction score and dissatisfaction scores by domain were constructed, according to the following steps:

1. Group items according to domains (social environment, economic environment, physical environment, healthcare, and infrastructures/services; Table 1). Items that did not clearly fit these domains (e.g., are you satisfied with the life you lead) were not included (*n* = 5). Groupings were made after discussion between the coauthors. Besides, we examined the robustness of the results to alternate specifications of the scores by serially excluding items and recalculating the score (results remained unchanged, data not shown).
2. Calculate the mean proportion of the dissatisfied and very dissatisfied for each of the 50 included items. We used the mean proportions of the five surveys instead of using a single survey to capture the average ratings of each city. For items with a scale ranging from totally agree to totally disagree we summed the proportion of residents that totally agree/agree or those that totally disagree/disagree depending on whether the issue was detrimental or beneficial.
3. Classify the obtained proportion into quintiles according to obtain a punctuation ranging from 1 to 5 (1 = least dissatisfied … 5 = most dissatisfied).
4. Average the punctuations of the items to obtain a global dissatisfaction score and then average according to domain to obtain dissatisfaction scores by domain for each city. This method of generating punctuations and scores has been employed elsewhere (Ribeiro et al., 2015; Hoffimann et al., 2017) and allow to generate a measure of dissatisfaction based on rank position of each city in the sample distribution.

Because not all cities had complete information on both residents' dissatisfaction and mortality, we included only those with complete information, a total of 74 European cities from 29 countries pertaining to four European regions—Western (*n* = 32 cities), Southern (*n* = 15), Northern (*n* = 10), and Eastern Europe (*n* = 17) (EUROVOC, 2016). These four regions are characterized by different political, socioeconomic and cultural environments (Vågerö, 2010).

Data on mortality and population were obtained from the Eurostat database for the latest year available (mostly 2013). Total counts of deaths for each city were obtained, as well as deaths by sex and age group for the EU-28, which was used as reference to calculate Standardized Mortality Ratios (SMR) and corresponding 95% Confidence Intervals (95% CI).

### Statistical analysis

Generalized Linear Models (Gaussian) were used to estimate the association between the SMR in each city and residents' dissatisfaction scores. We fitted four different models. First, we measured the bivariate associations between each dissatisfaction score, European region and the SMR (Model 0). Model 1 includes only the SMR and the variable “European Region.” In Model 2, dissatisfaction scores were added simultaneously, and successively removed, so that only predictors that made a significant unique contribution were retained. Finally, Model 3 is the same as Model 2, but adjusted for European regions.

It is important to refer that the role of the variable “European Region” was explored because it was clearly associated with both mortality and dissatisfaction and it was not in the pathway between the two, so that it acted as a confounder of the association we aimed to estimate. Besides, to exclude the hypothesis “region” could be a moderator, an interaction term was added to the final model, but it was not statistically significant (*p* = 0.269).

A significance level of 0.05 was used. A Gaussian model was used instead of Poisson's because the SMR were normally distributed and the counts of the deaths were too over-dispersed to run a Poisson or even a negative Binomial model (Kwan, 2014).

## Results

**Figure 1:**
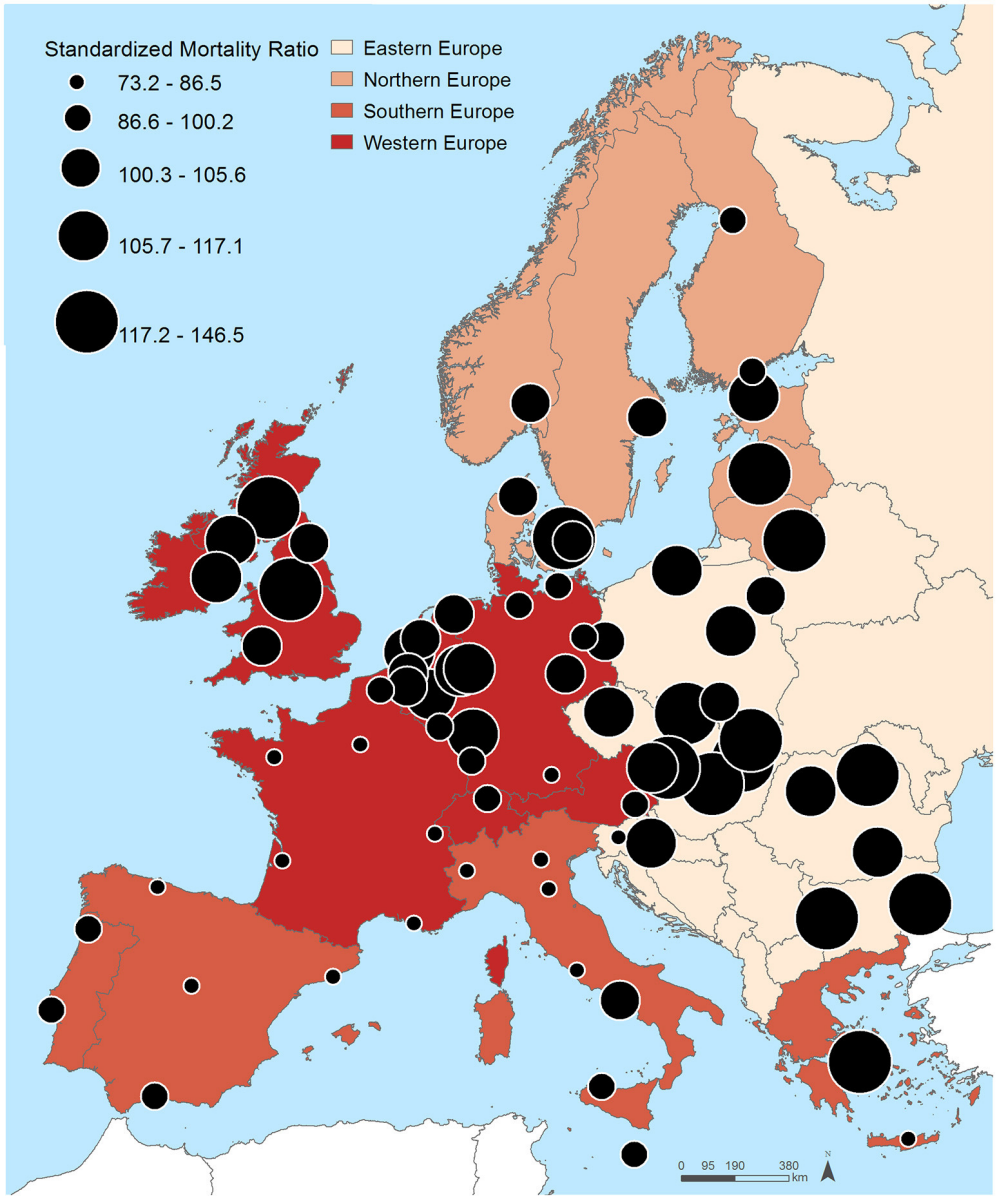
Spatial distribution of the standardized mortality ratios across the 74 included cities.

Figure 1 and Table 2 show the distribution of the SMR for the 74 cities included in this study. Twenty-one cities registered SMR significantly lower than 100 (the EU-28 average) and 37 SMR significantly higher than 100. As observable in Figure 1, there was a clear Northeast-Southwest division of the SMR, with the highest SMR predominantly found in Eastern and Northern European cities—Miskolc (146.5; 95% CI 140.6–152.6), Riga (136.3; 133.5–139.1), Sofia (133.8; 131.6–136.1), Copenhagen (132.4; 128.4–136.4), Burgas (131.1; 125.6–136.7) and Ostrava (130.2; 125.8–134.7)—and the lowest in Western and Southern European cities—Paris (73.1; 72.0–74.4), Madrid (74.0; 73.1–74.9), Rennes (75.7; 72.8–78.8), Barcelona (79.6; 78.3–80.8), and Heraklion (80.7; 75.5–86.0).

**Table 2:** Standardized Mortality Ratios (SMR) and 95% Confidence Intervals (IC 95%) in the 74 European cities (ascending order).

**City (country, region)**	**SMR**	**IC 95%**
Paris (FR, W)	73.2	72.0–74.4
Madrid (ES, S)	74.0	73.1–74.9
Rennes (FR, W)	75.7	72.8–78.8
Barcelona (ES, S)	79.6	78.3–80.8
Heraklion (EL, S)	80.7	75.5–86.0
Verona (IT, S)	81.4	78.3–84.5
Bordeaux (FR, W)	81.6	79.5–83.1
Geneva (CH, W)	83.8	79.6–88.2
Rome (IT, S)	83.8	82.8–84.8
Bologna (IT, S)	83.8	81.4–86.3
Munich (DE, W)	84.4	82.9–86.0
Marseille (FR, W)	84.4	82.7–86.1
Turin (IT, S)	84.5	82.9–86.2
Ljubljana (SI, E)	84.6	81.1–88.1
Oviedo (ES, S)	86.5	82.9–90.1
Braga (PT, S)	86.8	81.9–92.0
Graz (AT, W)	88.9	85.1–92.8
Oulu (FI, N)	90.4	84.8–96.3
Málaga (ES, S)	90.8	88.2–93.5
Lisbon (PT, S)	90.9	88.8–93.1
Strasbourg (FR, W)	91.6	88.6–94.6
Luxembourg (LU, W)	93.5	86.4–101.0
Rostock (DE, W)	94.4	90.6–98.4
Palermo (IT, S)	95.7	93.4–98.1
Lille (FR, W)	96.7	94.6–98.7
Zurich (CH, W)	97	93.8–100.3
Valletta (MT, S)	99.3	94.7–104.1
Helsinki (FI, N)	99.5	96.7–102.3
Berlin (DE, W)	99.7	98.6–100.7
Hamburg (DE, W)	100.2	98.7–101.7
Leipzig (DE, W)	100.3	97.7–102.8
Kraków (PL, E)	101.1	98.8–103.5
Bialystok (PL, E)	101.1	97.2–105.2
Newcastle (UK, W)	101.5	97.4–105.8
Stockholm (SE, N)	102.1	99.8–104.6
Groningen (NL, W)	102.5	97.1–108.2
Aalborg (DK, N)	102.7	98.0–107.5
Naples (IT, S)	103.3	101.3–105.4
Malmö (SE, N)	104.1	100.3–108.1
Oslo (NO, N)	104.6	101.5–107.7
Brussels (BE, W)	104.8	102.7–106.9
Frankfurt (DE, W)	104.8	97.6–112.5
Cardiff (UK, W)	105.2	101.3–109.1
Antwerp (BE, W)	105.3	102.4–108.1
Amsterdam (NL, W)	105.6	102.8–108.4
Warsaw (PL, E)	105.6	104.1–107.1
Tallinn (EE, N)	107.2	104.0–110.6
Gdansk (PL, E)	108.3	105.3–111.5
Kaiserslautern (DE, W)	109	102.7–115.4
Vienna (AT, W)	109.6	108.0–111.3
Dortmund (DE, W)	110.3	107.7–112.9
Prague (CZ, E)	110.5	108.5–112.4
Bucharest (RO, E)	111.9	110.4–113.4
Essen (DE, W)	112.6	110.1–115.2
Rotterdam (NL, W)	112.9	110.0–115.9
Cluj-Napoca (RO, E)	113.4	109.3–117.6
Dublin (IE, W)	114.6	111.2–118.2
Belfast (UK, W)	115.1	110.8–119.6
Zagreb (HR, E)	115.6	113.2–118.1
Liège (BE, W)	117.1	113.7–120.6
Bratislava (SK, E)	117.8	114.2–121.5
Piatra Neamt (RO, E)	120.8	113.5–128.3
Manchester (UK, W)	122	118.0–126.1
Kosice (SK, E)	122.3	117.1–127.7
Budapest (HU, E)	125.1	123.4–126.7
Vilnius (LT, N)	129.2	125.9–132.6
Ostrava (CZ, E)	130.2	125.8–134.7
Burgas (BG, E)	131.1	125.6–136.7
Copenhagen (DK, N)	132.4	128.4–136.4
Sofia (BG, E)	133.8	131.6–136.1
Glasgow (UK, W)	135.9	132.6–139.3
Riga (LI, N)	136.3	133.5–139.1
Athens (EL, S)	145.7	142.9–148.5
Miskolc (HU, E)	146.5	140.6–152.6

*AT, Austria; BE, Belgium; BG, Bulgaria; CH, Switzerland; CZ, Czech Republic; DE, Germany; DK, Denmark; EE, Estonia; EL, Greece; ES, Spain; FI, Finland; FR, France; HR, Croatia; HU, Hungary; IT, Italy; LI, Lithuania; LT, Latvia; LU, Luxembourg; MT, Malta; NL, Netherlands; NO, Norway; PL, Poland; PT, Portugal; RO, Romania; SI, Slovenia; SK, Slovakia; SE, Sweden; UK, United Kingdom; W, Western Europe; N, Northern Europe; S, Southern Europe; E, Eastern Europe*.

**Figure 2:**
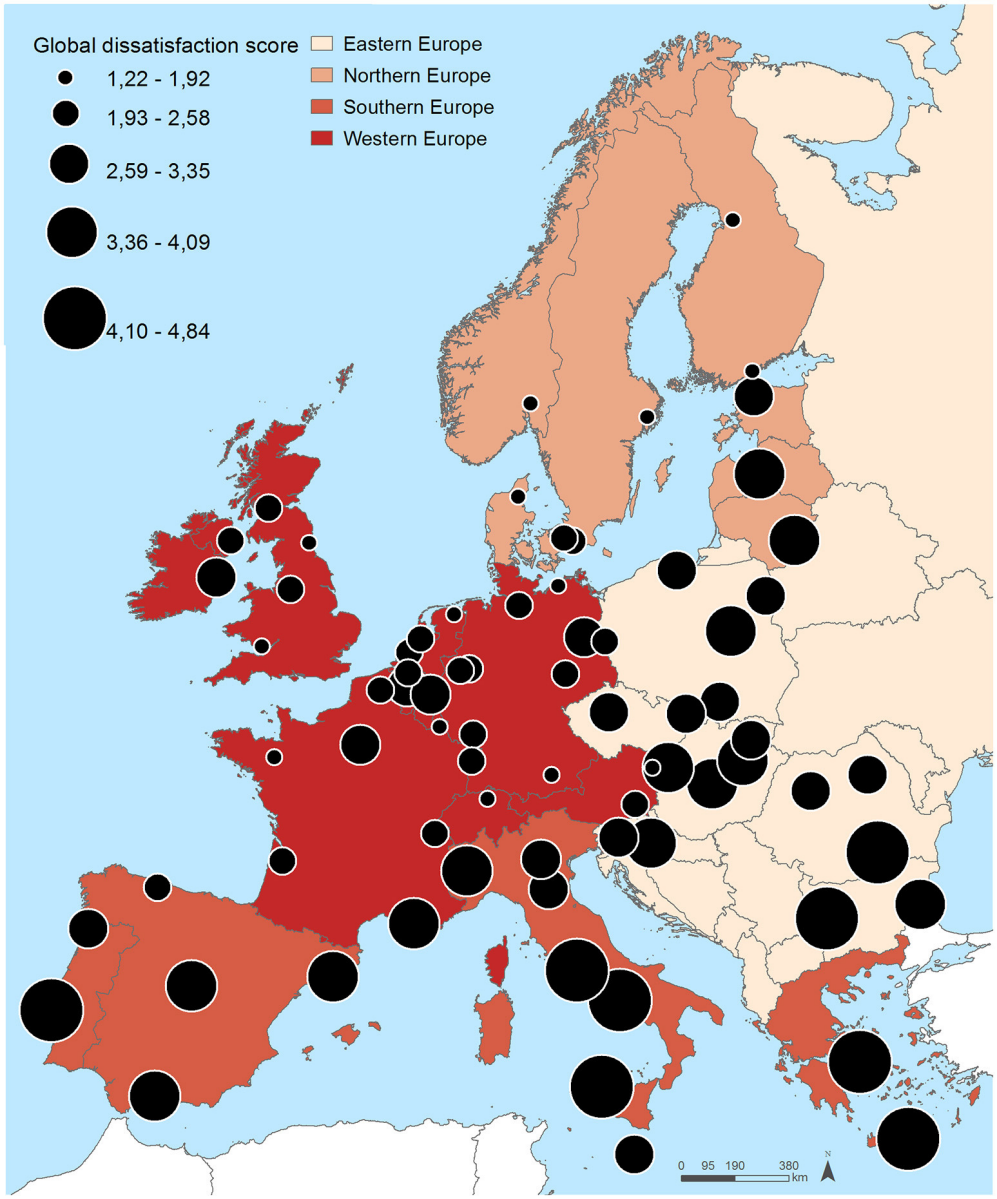
Spatial distribution of the global dissatisfaction scores across the 74 included cities.

Figure 2 and Table 3 show the distribution of the residents' dissatisfaction scores. Large geographical differences were also found in the residents' dissatisfaction scores. Eastern and some Southern European cities (mean global dissatisfaction score 3.48 and 3.82, respectively) tended to have higher dissatisfaction scores, contrasting with Western and Northern European cities (mean global dissatisfaction score 2.25 and 2.35, respectively). The five cities with highest global dissatisfaction score were Naples (score of 4.84), Athens (4.84), Rome (4.80), Palermo (4.65), and Sofia (4.45), whereas the lowest were observed in Luxembourg (1.66), Newcastle (1.50), Munich (1.46), Aalborg (1.38), and Zurich (1.31).

**Table 3:** Residents' dissatisfaction scores according to domain in the 74 European cities (ascending order according global dissatisfaction score).

**City (country, region)**	**Global**	**Economy**	**Physical**	**Healthcare**	**Infrastructures/services**	**Social**
Zurich (CH, W)	1.31	1.80	1.00	1.00	1.63	1.14
Aalborg (DK, N)	1.38	1.00	1.30	1.67	1.50	1.43
Munich (DE, W)	1.46	1.67	1.30	1.00	1.93	1.43
Newcastle (UK, W)	1.50	1.75	1.29	1.00	1.67	1.80
Luxembourg (LU, W)	1.66	1.83	1.10	1.67	2.29	1.43
Cardiff (UK, W)	1.73	1.50	1.30	2.00	1.71	2.14
Oulu (FI, N)	1.77	1.67	1.40	2.33	1.86	1.57
Rennes (FR, W)	1.83	2.67	1.20	1.00	2.00	2.29
Stockholm (SE, N)	1.85	1.67	1.80	2.00	2.21	1.57
Oslo (NO, N)	1.85	2.40	2.00	1.00	2.13	1.71
Rostock (DE, W)	1.87	2.17	1.20	2.00	2.55	1.43
Wien (AT, W)	1.90	1.83	1.60	1.33	2.00	2.71
Helsinki (FI, N)	1.92	2.00	1.70	2.67	1.50	1.71
Leipzig (DE, W)	1.95	2.33	1.40	1.67	2.36	2.00
Graz (AT, W)	1.97	1.83	2.50	1.00	2.36	2.14
Geneva (CH, W)	1.99	2.80	1.80	1.00	1.63	2.71
Copenhagen (DK, N)	2.02	1.67	3.00	1.67	2.36	1.43
Bordeaux (FR, W)	2.05	3.50	1.70	1.00	2.07	2.00
Belfast (UK, W)	2.08	2.17	1.80	2.00	2.14	2.29
Hamburg (DE, W)	2.12	2.33	1.60	2.00	2.64	2.00
Oviedo (ES, S)	2.12	2.67	1.50	2.00	2.57	1.86
Malmö (SE, N)	2.12	1.67	1.80	3.00	1.86	2.29
Amsterdam (NL, W)	2.17	2.50	2.60	1.33	2.00	2.43
Antwerp (BE, W)	2.20	2.00	3.00	1.00	1.71	3.29
Strasbourg (FR, W)	2.25	3.33	2.40	1.00	1.79	2.71
Rotterdam (NL, W)	2.25	2.00	3.00	1.33	1.79	3.14
Essen (DE, W)	2.27	1.50	2.60	1.67	3.00	2.57
Kaiserslautern (DE, W)	2.30	4.00	1.40	2.00	2.38	1.71
Manchester (UK, W)	2.34	2.17	2.30	2.00	2.64	2.57
Dortmund (DE, W)	2.35	2.17	2.20	1.67	3.00	2.71
Glasgow (UK, W)	2.35	2.67	2.60	2.00	2.36	2.14
Frankfurt (DE, W)	2.56	3.00	2.00	2.00	3.78	2.00
Lille (FR, W)	2.58	3.67	2.60	1.00	2.21	3.43
Ljubljana (SI, E)	2.66	2.83	2.40	3.00	2.93	2.14
Bialystok (PL, E)	2.74	3.33	1.30	4.33	2.57	2.14

*AT, Austria; BE, Belgium; BG, Bulgaria; CH, Switzerland; CZ, Czech Republic; DE, Germany; DK, Denmark; EE, Estonia; EL, Greece; ES, Spain; FI, Finland; FR, France; HR, Croatia; HU, Hungary; IT, Italy; LI, Lithuania; LT, Latvia; LU, Luxembourg; MT, Malta; NL, Netherlands; NO, Norway; PL, Poland; PT, Portugal; RO, Romania; SI, Slovenia; SK, Slovakia; SE, Sweden; UK, United Kingdom; W, Western Europe; N, Northern Europe; S, Southern Europe; E, Eastern Europe*.

Very similar patterns were observed for the domains of dissatisfaction. Dissatisfaction with the economic environment (i.e., unemployment, poverty, ability to make ends meet) was highest in Lisbon (5.00), Naples (4.84), Rome (4.80), Athens (4.84) and Palermo (4.65) and lowest in Aalborg (1.00), Cardiff (1.50), Essen (1.50), Munich (1.67), and Oulu (1.67). Concerning the physical environment (i.e., green spaces, noise, pollution), higher dissatisfaction scores were observed in Rome (4.90), Bucharest (5.00), Sofia (5.00), Athens (5.00) and Naples (5.00), and the lowest in Zurich (1.00), Luxembourg (1.10), Rostock (1.20), Rennes (1.20), and Newcastle (1.29). Healthcare services were rated more poorly (5.00) in Naples, Athens, Sofia, Bucharest, Rome, Palermo, Warsaw, Burgas, Riga, Vilnius, Cluj-Napoca, Gdansk, and Pietra Neamt, whereas Zurich, Rennes, Newcastle, Munich, Bordeaux, Geneva, Oslo, Strasbourg, Graz, Lille, Antwerp, Liege, and Brussels exhibited lower dissatisfaction levels (1.00). Higher residents' dissatisfaction with infrastructures/services (i.e., transport, schools, cultural facilities) were observed in Madrid (4.36), Palermo (4.43), Rome (4.43), Naples (4.50) and Athens (4.71) and lower in Aalborg (1.50), Helsinki (1.50), Zurich (1.00), Geneva (1.00), and Newcastle (1.00). And, finally, the dissatisfaction with the social environment (i.e., trust safety, integration) was higher in Palermo (4.57), Turin (4.86), Naples (4.86), Rome (5.00), and Athens (5.00), and lesser in Zurich (1.14), Aalborg (1.43), Munich (1.43), Luxembourg (1.43), Copenhagen (1.43) and Rostock (1.43).

**Figure 3:**
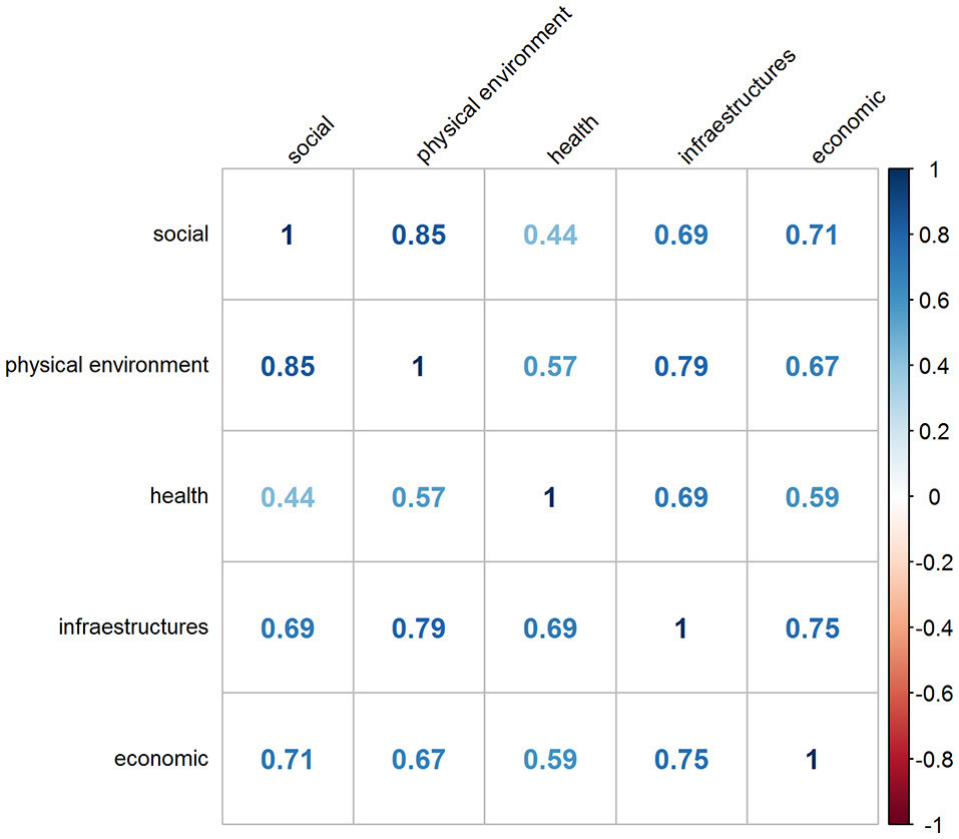
Correlations between the domains of residents' dissatisfaction.

It is important to note that all domains of dissatisfaction were moderately-highly correlated, and particularly strong was the correlation between dissatisfaction with physical environment and social environment (*r* = 0.85; Figure 3), meaning that generally urbanites tend to be simultaneously dissatisfied with numerous aspects of the city environment.

Table 4 shows the associations between residents' dissatisfaction scores and mortality. In Model 0, we looked for the bivariate associations between each dissatisfaction score, region and SMR and we found that only the dissatisfaction score with healthcare (β = 0.286; 95% CI 0.065, 0.507) was significantly associated with the SMR, although all other regression coefficients were positive, indicating that increased dissatisfaction with cities' attributes was associated with increased mortality. European region was also significantly associated with mortality: compared with Eastern Europe, Southern (−1.476; −2.080, −0.871) and Western European cities (−0.896; −1.408, −0.384) presented significantly lower levels of mortality.

**Table 4:** Association between residents' dissatisfaction scores and standardized mortality ratio in the 74 cities.

**Variables**	**Model 0 (β and 95% CI)**	**Model 1 (β and 95% CI)**	**Model 2 (β and 95% CI)**	**Model 3 (β and 95% CI)**
**DISSATISFACTION SCORES**				
Economic	0.007 (−0.224; 0.238)		–	–
Physical environment	0.171 (−0.057; 0.399)		–	–
Healthcare	**0.286 (0.065; 0.507)**		**0.286 (0.065; 0.507)**	**0.334 (0.030; 0.639)**
Infrastructures/services	0.093 (−0.137; 0.323)		–	–
Social	0.142 (−0.087; 0.370)		–	**0.239 (0.015; 0.464)**
**EUROPEAN REGION**				
Eastern	Ref	Ref	–	Ref
Northern	−0.326 (−1.006; 0.354)	−0.326 (−1.006; 0.354)	–	0.196 (−0.506; 0.899)
Southern	**−1.476 (−2.080; −0.871)**	**−1.476 (−2.080; −0.871)**	–	**−1.510 (−2.096; −0.923)**
Western	**−0.896 (−1.408; −0.384)**	**−0.896 (−1.408; −0.384)**	–	−0.153 (−0.859; 0.553)
Variance explained (%)	–	**24.2**	**6.9**	**33.7**

*Model 0, univariable; Model 1, multivariable, not adjusted for European region; Model 2, multivariable, adjusted for European region*.

*In bold, statistical significant coefficients*.

*β, standardized regression coefficient and 95% Confidence Intervals (CI)*.

Model 1 only included mortality and the European regions. The proportion of variance explained by this simple model was nevertheless 24.2%.

In Model 2, to take into account the inter-correlation between the five dissatisfaction scores, we entered all dissatisfaction scores in the model and kept only those significantly associated with the SMR. We observed that only the healthcare dissatisfaction score (0.286; 0.065, 0.507) was independently and significantly associated with the SMR, i.e., the higher the dissatisfaction levels of the residents with health aspects the higher the mortality. All the other dissatisfaction scores lost statistical significance, probably due to the high degree of inter-correlation between them, as depicted in Figure 3. The proportion of variance explained by Model 2 was very low: 6.9%, much lower than when only European regions were considered.

Adjusting for the European regions (Model 3), dissatisfaction with healthcare (0.286; 0.065, 0.507) and with social environment (0.286; 0.065, 0.507) were both found to be significantly and positively associated with mortality. At the same time, the proportion of variance explained by the model increased to 33.7%.

## Discussion

This study was one of the first addressing the health status of European cities. Grounded on a set of Europe-wide Quality of Life Surveys, we were able to explore the link between residents' dissatisfaction with the city and health. We found profound differences in mortality across 74 cities in Europe, with the highest risk of death generally found in Eastern and Northern Europe and the lowest in the South and Western European cities. Residents' dissatisfaction levels varied greatly as well, and were generally higher in Eastern Europe and in some Southern European cities. We found a significant association between city levels of mortality and residents' dissatisfaction with healthcare services and social environment.

The Southwest-Northeast division of health in Europe was a constant in this study. The East-West divide of Europe has been extensively reported elsewhere (Vågerö, 2010). Similarly, the comparatively unexpectedly poor performance in life expectancy gains in some Northern European countries has also been matter of discussion (Juel et al., 2000). This panorama supports the idea that, although cities share some characteristics, both detrimental (pollution, segregation, crowding) and beneficial (concentration of employment, equipment, goods), truth is they seem to reproduce the health status of the whole country making them a kind of a barometer of national health. The Southwest-Northeast division of Europe was also observed for the dissatisfaction levels of the residents, although in Southern European cities a rather large proportion of the residents did also rate poorly several aspects of the city functioning and resources. Real-life problems in city functioning and resources, but also cross-national cultural differences might explain these differences. For instance, when it comes to self-reported health, studies observed cross-national and cultural differences in self-assessed health; generally southern European respondents tended to perceived more poorly their health status than their Scandinavian counterparts (Jürges, 2007).

We found that after removing the effect of the European region (a latent variable that summarize the tremendous structural and cultural differences between European countries/regions), citizen's dissatisfaction levels with key aspects of the urban resources and functioning—healthcare and social environment—were associated to mortality levels of the cities. Evidence of an association between residents' satisfaction and health outcomes and well-being can be found in the literature. Hogan and colleagues, in a four-city study, have precisely observed an association between residents' ratings about city performance/amenities and their happiness levels (Hogan et al., 2016). Shiue, in Taiwan, found that satisfaction with neighborhood environment was related with self-rated health (Shiue, 2012). And, finally, perceived neighborhood safety and social environment have been consistently associated with numerous health outcomes (Wen et al., 2006; Kim et al., 2013; Polling et al., 2014; Assari et al., 2015).

Apart from the previously mentioned studies, which addressed subjective feelings about the city's performance, the health-impact of the urban environment has been essentially evaluated through objective assessments. Interestingly, our findings corroborate those drawn from studies that used objective measures. This highlights the importance of keep conducting large surveys, as the ones employed in the present study, and of using these data as a complement of objective information. In addition, using perceived/subjective measures offers a number of advantages. Certain issues, such as aesthetics, safety, and disorder, satisfaction with the way services work and/or feelings and attitudes, are difficult to capture using objective assessments (McCrea et al., 2006). Moreover, subjective measures capture the “human perception of space” (i.e., place) improving our the understanding of the urban environment (McCrea et al., 2006; Kothencz et al., 2015). Putting in other words, the objective attributes of the city's environment, once they have been evaluated by the individual (and the personal characteristics and experience they carry), become subjective, and lead to a certain degree of satisfaction (John, 1987; Amérigo and Aragonés, 1997).

Regarding the impact of social environment, our results corroborate the literature reporting that social support and social connectedness networks are very important, not only as a complement to the formal healthcare system, but also as a protection against the adversities inherent of being ill, being poor and being alone (Seeman, 1996). Indeed most of the studies that addressed whether residential satisfaction affected health outcomes, point toward the same direction. Perceived neighborhood safety and social environment have been associated with self-rated health (Wen et al., 2006; Kim et al., 2013; Assari et al., 2015), health-related quality of life (Parra et al., 2010), mental illness (Polling et al., 2014), and stroke risk (Kim et al., 2013). And, importantly, some of these studies confirmed this association remained significant after accounting for objective measures (Wen et al., 2006; Kim et al., 2014) and others found that indeed perceived measures of the social environment, rather than objective ones, show a larger association over the studied outcomes (Polling et al., 2014). It is also interesting to note that our results do also show a particularly strong correlation between social and physical environment that has also been reported elsewhere; apparently, health-supportive physical environments influence the social capital of the places, by reducing social inequalities (Mitchell and Popham, 2008), and by enhancing social interaction and social inclusion (Maas et al., 2009).

When it comes to healthcare, although the questions from Urban Audit were comparatively fewer and somehow vague, the answers might provide an idea on how satisfied patients were with healthcare services. In our study, we found a significant association with mortality—higher mortality rates were observed in cities with higher dissatisfaction scores with healthcare—indicating that, as other suggested, the systematic evaluation of patient satisfaction might provide useful information on patients' experiences that can contribute for improving the performance of healthcare services and the quality of care provided to the patients (Browne et al., 2010).

Several studies and reports support the health benefits of high patient satisfaction; satisfied patients may have increased treatment adherence and better health outcomes (Chue, 2006; Glickman et al., 2010; Zgierska et al., 2014). Also suggesting that satisfaction with healthcare is critical for the population well-being, Hogen and colleagues observed that the effect of healthcare performance on well-being and happiness was significant for all groups and not only for those over 65 years, contrasting with most of the studied satisfaction domains that did only associate with happiness in specific age groups (Hogan et al., 2016).

In our study, we did not find a significant association between dissatisfaction levels with the physical environment and mortality, a topic that numerous studies have explored by looking at the impact of air pollution exposure. Multiple polled and meta-analysis studies showed air pollution is directly associated with mortality, cardiorespiratory diseases and allergies (Beelen et al., 2014). Access to greenspace and noise exposure did also seem to be associated with numerous health outcomes: overall mortality (Barceló et al., 2016; Gascon et al., 2016), mental health (Lee and Maheswaran, 2011), and behaviors, such as physical activity (Cohen et al., 2007). More recently, increasing attention has been given to the contact with nature and water within urban contexts (Hartig and Kahn, 2016). According to numerous studies conducted in different countries, the human interaction with natural environment provides opportunities for relaxation, enhances connections between urban inhabitants and the biosphere, and promotes subjective well-being and happiness (MacKerron and Mourato, 2013; Marketta et al., 2015; Hartig and Kahn, 2016; Samuelsson et al., 2018).

Yet, several other studies have found that, compared to other aspects, namely socioeconomic conditions and access to healthcare, the physical environment play a less important role (Hood et al., 2016). Moreover, some investigations have shown that the exposure to harmful physical environments may be more strongly associated with specific causes of death, namely cancer, and not so strongly with overall mortality (Ribeiro et al., 2015). Regrettably, in our study we were not able to differentiate the causes of death. Note that, as previously mentioned, physical, and social environment dissatisfaction scores were very correlated, which means that these two kinds of deprivation tend to happen simultaneously, making it difficult to separate their effects.

Although several studies suggest that availability of destinations, services and good-quality infrastructures bring numerous health benefits (Frumkin, 2002), from obesity prevention (Sarkar et al., 2017) to feelings of well-being (Marketta et al., 2015), in our study, no significant association between dissatisfaction with infrastructures/services and mortality levels was observed. Similarly, we found that perceived economic environment was not significantly related with mortality in European cities. This finding deserves further validation, as the absence of an association does not imply they are uncorrelated. The fact we did not find a significant link between urban mortality and resident's ratings about the economic circumstances might be related with the specificity of our data and of our study area. The link between health and socioeconomic individual or group characteristics is probably one of the oldest and solidest findings in public health. But, these findings have been mostly drawn on objective indicators of socioeconomic circumstances (personal income or area level income, unemployment rates, occupation, etc.), which might not necessarily reflect the people's feelings about their and cities' economic problems. Some authors argue that income inequality, the discrepancy in income between population groups, rather than income, the population average, might be particularly important for health, being associated with higher disparities and lower longevity and life expectancy (Wilkinson, 1992; Truesdale and Jencks, 2016). The commonly used indicators of economic environment evaluate the amount of disposable income and material resources per capita, which despite being undoubtedly essential, turn a blind eye to people's beliefs about the fairness of income distribution, perceptions of their own income and their ability to make ends meet. These aspects are particularly important in the definition of deprivation. Deprivation refers to unmet need, which is caused by a lack of all kinds of resources, rather than financial needs alone and it can also be categorized as objective or subjective (Townsend, 1979, 1987; Guillaume et al., 2016). Objective deprivation is perceived collectively or socially and is registered in the census; subjective deprivation is individually perceived and is assessed by questionnaire in specific surveys (Townsend, 1979, 1987; Guillaume et al., 2016). Finally, it is also important to highlight that, in general, cities are characterized for being economically dynamic places even in more disadvantaged regions/countries, which might mean that what would distinguish a city from another might be aspects unrelated with economic circumstances (Hogan et al., 2015, 2016), as the ones we identified.

Our study presents some limitations that deserve further discussion. Firstly, we have focused on a pre-selected set of large cities leaving behind medium and small urban settings, which hold a significant amount of the European population. Then, our results might not be valid for these medium and small urban settings. Secondly, we have also focused on a single indicator of population health, mortality. A study of such nature would be improved by including other measures of health status such as preventable mortality or healthy life expectancy, unfortunately not available at city-level. Additionally, we assumed a single value of mortality of each city, despite knowing that European cities tend to exhibit a rather large within city heterogeneity in health outcome (Marí-Dell'Olmo et al., 2015) that an overall measure might not capture. The ecological design constitutes another important limitation, as we cannot guarantee the observed associations occur at individual-level too. Yet, notice that a dozen cross-sectional studies, conducted at individual-level, reported a significant relationship between satisfaction and individual health status (Wen et al., 2006; Shiue, 2012; Kim et al., 2013; Polling et al., 2014; Assari et al., 2015). Finally, the present study relied on a relatively small dataset and we could not confront our results with the ones that would be obtained by using objective measures about the urban environment, which would be ideal, since they are non-overlapping measures complementing each other (Nyunt et al., 2015).

Study strengths should be highlighted too. Our study provides a global view of the health status of European urbanities and it is the first establishing a link between residents' ratings about several aspects of city functioning and resources and objective measures of health. Because we grounded our study on Urban Audit Database, our geographical units and indicators are directly comparable, which strengthens our study. Our findings may also be important for city planners as they showed a relationship between citizen's perceptions about certain urban attributes and mortality, suggesting that measuring citizen's satisfaction with the urban environment might aid in the construction of healthier cities.

In conclusion, we revealed large inequalities in health between European cities and we found a significant association between city levels of mortality and residents' dissatisfaction with certain urban features, suggesting subjective information can be also used to comprehend the health of the urbanities. At a city-level, dissatisfaction with the healthcare services (care provided by doctors and hospitals) and social environments (trust, networks) seemed to be detrimental factors to health across European cities. So, although much more attention has been given to traditional economic and material determinants of health, these complementary aspects should not be disregarded by the local governments.

## Author contributions

AR designed the study, performed the statistical analysis and drafted the manuscript. SF and HB supervised the research, contributed to the interpretation of results and helped to draft the manuscript. All authors read and approved the final manuscript.

### Conflict of interest statement

The authors declare that the research was conducted in the absence of any commercial or financial relationships that could be construed as a potential conflict of interest.
